# Renoprotective Potential of Nateglinide in an Acute Kidney Injury Model

**DOI:** 10.3390/ijms27073021

**Published:** 2026-03-26

**Authors:** Senanur Ilıkça, Samet Öz, Güldeniz Şekerci, Aslı Taşlıdere, Suat Tekin

**Affiliations:** 1 Department of Physiology, Faculty of Medicine, Inonu University, 44280 Malatya, Turkey; senanurturkol1@gmail.com (S.I.); guldenizsekerci@gmail.com (G.Ş.); 2 Department of Veterinary Medicine, School of Health Services, Osmaniye Korkut Ata University, 80000 Osmaniye, Turkey; sametoz@osmaniye.edu.tr; 3 Department of Histology and Embryology, Faculty of Medicine, Inonu University, 44280 Malatya, Turkey; aslicetin1@yahoo.com

**Keywords:** acute kidney injury, apoptosis, inflammation, ischemia/reperfusion, nateglinide, oxidative stress

## Abstract

Nateglinide (Nat) is an oral antidiabetic agent of the meglitinide class that has been reported to exert protective effects beyond glycemic control, particularly against oxidative stress and inflammation. Since oxidative stress and inflammation play a key role in the pathogenesis of acute kidney injury (AKI), especially following ischemia/reperfusion (I/R), this study aimed to evaluate the potential renoprotective effects of Nat in a rat model of I/R-induced AKI. Forty male Sprague Dawley rats were randomly divided into four groups (*n* = 10): Control, I/R, I/R + Nat (50 mg/kg), and I/R + Nat (100 mg/kg). Bilateral renal ischemia was induced by clamping renal arteries for 45 min, followed by 24 h of reperfusion. Nat was administered orally 1 h before ischemia. Renal levels of superoxide dismutase (SOD), catalase (CAT), glutathione (GSH), and thiobarbituric acid reactive substances (TBARSs) were assessed. Serum blood urea nitrogen (BUN), creatinine, tumor necrosis factor-α (TNF-α), and interleukin-1β (IL-1β) were also measured, and histopathological analyses were performed. Nat significantly increased renal antioxidant parameters and reduced TBARS levels. Moreover, Nat markedly decreased serum BUN, creatinine, TNF-α, and IL-1β levels compared with the I/R group (*p* < 0.05). Histopathology confirmed attenuated renal damage in Nat-treated groups (*p* < 0.0001). These results indicate that Nat confers significant renoprotection against renal I/R injury via suppression of oxidative stress and inflammation.

## 1. Introduction

Acute kidney injury (AKI) is a severe clinical condition characterized by a rapid decline in glomerular filtration rates, accumulation of metabolic waste products, and disturbances in fluid–electrolyte homeostasis [1]. In intensive care units, the mortality rate associated with AKI can exceed 50%, making it a major global health concern [2]. One of the most common etiological factors contributing to AKI is renal I/R injury. Renal I/R injury occurs in clinical conditions such as shock, sepsis, major surgical procedures, kidney transplantation, severe trauma, and renal vascular occlusions, and it represents a primary mechanism underlying AKI [3,4,5,6]. Ischemia refers to the deprivation of oxygen due to interruption of renal blood flow, whereas reperfusion further amplifies injury through the excessive generation of free radicals upon reoxygenation [7,8]. Multiple pathological processes—including cell injury (apoptosis, necrosis, and ferroptosis), oxidative stress, and inflammatory responses—are critically involved in the pathogenesis of I/R injury [9]. Following reperfusion, adhesion of leukocytes to activated endothelial cells results in peritubular capillary congestion and the release of inflammatory and oxidative mediators, thereby aggravating renal injury [10]. Leukocytes, particularly neutrophils, monocytes/macrophages, and lymphocytes, adhere to the endothelium through adhesion molecules and infiltrate interstitial and perivascular tissues [11]. These infiltrating cells release pro-inflammatory and oxidative mediators such as cytokines (e.g., TNF-α, IL-1β, and IL-6), chemokines, and reactive oxygen species (ROS), which intensify tissue injury by promoting apoptosis and necrosis, edema, microvascular dysfunction, and loss of renal function [12]. In light of this pathophysiology, pharmacological agents with antioxidant and anti-inflammatory properties have shown promising outcomes in experimental I/R models [13].

Nat is an oral antidiabetic agent that stimulates rapid and short-acting insulin secretion by closing ATP-sensitive K^+^ channels in pancreatic β-cells, and is primarily used for the control of postprandial hyperglycemia [14,15]. Although Nat is not structurally derived from classical meglitinides, it exhibits similar pharmacodynamic properties and is therefore therapeutically grouped with agents such as Repaglinide [16]. Recent experimental and clinical findings suggest that Nat not only regulates glycemia but also modulates oxidative stress and inflammatory pathways [17]. In newly diagnosed diabetic patients, Nat has been shown to reduce blood glucose levels, decrease insulin resistance, alleviate oxidative stress, and improve endothelial function [17]. Since high glucose levels in renal tubular epithelial cells increase oxidative stress and inflammatory activation, this biological background provides a rationale for investigating the antioxidant and anti-inflammatory potential of Nat in renal I/R injury [18].

However, studies evaluating the effects of Nat in renal I/R models are still limited. Therefore, the present study aimed to investigate the potential renoprotective effects of Nat in an experimental rat model of AKI induced by renal I/R. For this purpose, oxidative stress parameters (superoxide dismutase (SOD), catalase (CAT), glutathione (GSH), and thiobarbituric acid reactive substances (TBARSs)), renal function markers (blood urea nitrogen (BUN), and creatinine), and inflammatory mediators (tumor necrosis factor-alpha (TNF-α) and interleukin-1β (IL-1β)), as well as histopathological and immunohistochemical (caspase-3) findings, were comprehensively evaluated.

## 2. Results

In this study, I/R injury was induced by clamping both renal pedicles for 45 min followed by 24 h of reperfusion in Sprague Dawley rats. Nat was administered orally at doses of 50 mg/kg and 100 mg/kg one hour before ischemia induction. Detailed experimental procedures are described in Section 4.3.

### 2.1. Effects of Nateglinide on Renal Function Markers

**Figure 1:**
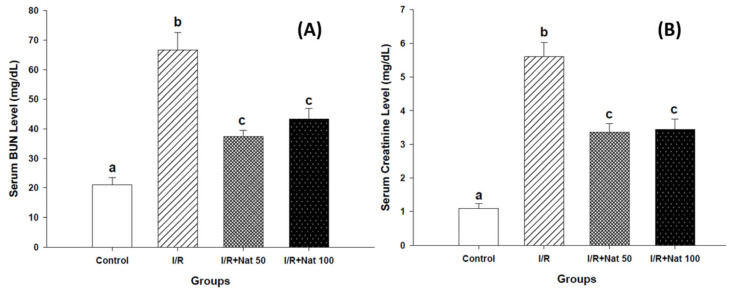
Effect of Nat on serum BUN (**A**) and creatinine (**B**) levels in renal I/R injury. Values are expressed as mean ± SD. Different superscript letters (a, b, c) indicate statistically significant differences (*p* < 0.05). I/R: ischemia/reperfusion group; Nat 50: nateglinide 50 mg/kg; Nat 100: nateglinide 100 mg/kg; BUN: blood urea nitrogen.

Serum BUN and creatinine levels are presented in Figure 1A,B. Both markers were significantly elevated in the I/R group compared to the Control group (*p* < 0.05). Nat administration at 50 and 100 mg/kg significantly attenuated these elevations (*p* < 0.05). Compared with the I/R group, Nat administration at 50 mg/kg reduced serum BUN levels by 45%, whereas the 100 mg/kg dose resulted in a reduction of 34%, indicating a marked improvement in renal function. Similarly, serum creatinine levels decreased by 39% and 38% in the I/R + Nat 50 and I/R + Nat 100 groups, respectively.

### 2.2. Effects of Nateglinide on Inflammatory Cytokines

**Figure 2:**
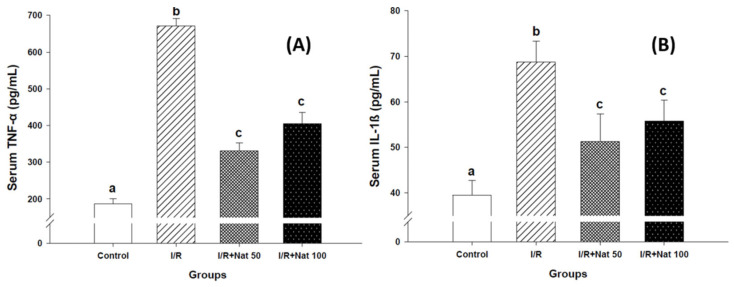
Effect of Nat on serum TNF-α (**A**) and IL-1β (**B**) levels in renal I/R injury. Values are expressed as mean ± SD. Different superscript letters (a, b, c) indicate statistically significant differences (*p* < 0.05). I/R: ischemia/reperfusion group; Nat 50: nateglinide 50 mg/kg; Nat 100: nateglinide 100 mg/kg; TNF-α: tumor necrosis factor-alpha; IL-1β: interleukin-1 beta.

Serum TNF-α and IL-1β levels are shown in Figure 2A,B. Both cytokines were significantly increased in the I/R group compared to controls (*p* < 0.05). Treatment with Nat (50 and 100 mg/kg) significantly reduced these inflammatory mediators (*p* < 0.05). Compared with the I/R group, Nat treatment reduced serum TNF-α levels by 50% and 39.6% in the I/R + Nat 50 and I/R + Nat 100 groups, respectively. Similarly, serum IL-1β levels decreased by 26.1% in the I/R + Nat 50 group and 18.8% in the I/R + Nat 100 group, confirming a substantial attenuation of the inflammatory response following Nat administration.

### 2.3. Effects of Nateglinide on Oxidative Stress Parameters

**Figure 3:**
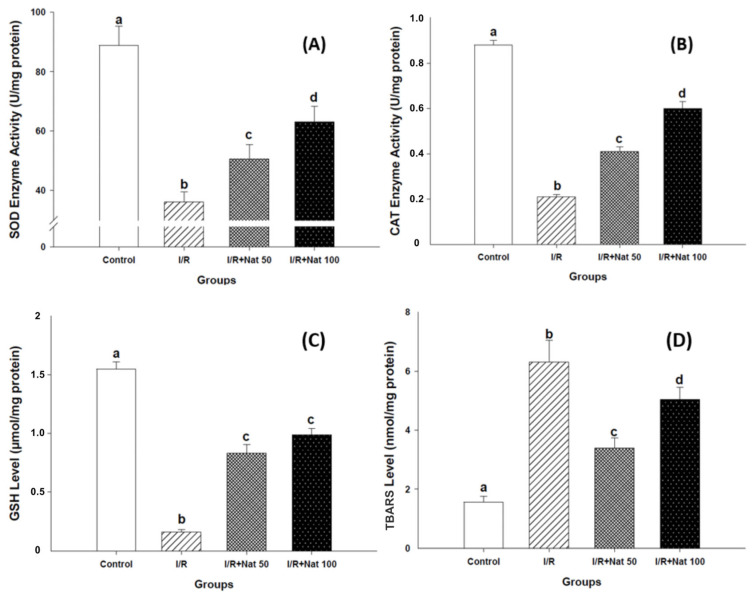
Effect of Nat on antioxidant enzyme activities (**A**–**C**) and lipid peroxidation (**D**) in renal I/R injury. SOD, CAT, and GSH activities were significantly decreased in the I/R group (**A**–**C**), while TBARS levels were markedly increased (**D**). Nat administration significantly improved antioxidant enzyme activities and reduced lipid peroxidation in a dose-dependent manner. Values are expressed as mean ± SD. Different superscript letters (a, b, c, d) indicate statistically significant differences between groups (*p* < 0.05). I/R: ischemia/reperfusion group; Nat 50: nateglinide 50 mg/kg; Nat 100: nateglinide 100 mg/kg; SOD: superoxide dismutase; CAT: catalase; GSH: glutathione; TBARS: thiobarbituric acid reactive substances.

In the Control group, SOD, CAT, and GSH levels were highest, whereas TBARS levels were the lowest. In the I/R group, antioxidant parameters showed significant decreases, accompanied by a marked increase in TBARS levels (*p* < 0.05). Nat treatment at 50 and 100 mg/kg significantly increased SOD, CAT, and GSH levels while significantly decreasing TBARS levels (*p* < 0.05). The 100 mg/kg dose was associated with the highest antioxidant activities and the greatest reduction in lipid peroxidation. Antioxidant results are shown in Figure 3A–C, and TBARS levels are shown in Figure 3D. Compared with the I/R group, Nat treatment increased SOD activity by 38.9% and 75% in the I/R + Nat 50 and I/R + Nat 100 groups, respectively. CAT activity showed a marked elevation of 95.2% in the I/R + Nat 50 group and 185.7% in the I/R + Nat 100 group. Similarly, renal GSH levels increased by 453.3% and 560.0% following administration of 50 mg/kg and 100 mg/kg Nat, respectively. In contrast, lipid peroxidation was significantly attenuated, with TBARS levels reduced by 46.9% in the I/R + Nat 50 group and 20.3% in the I/R + Nat 100 group compared with the I/R group.

### 2.4. Histopathological and Immunohistochemical Findings

**Figure 4:**
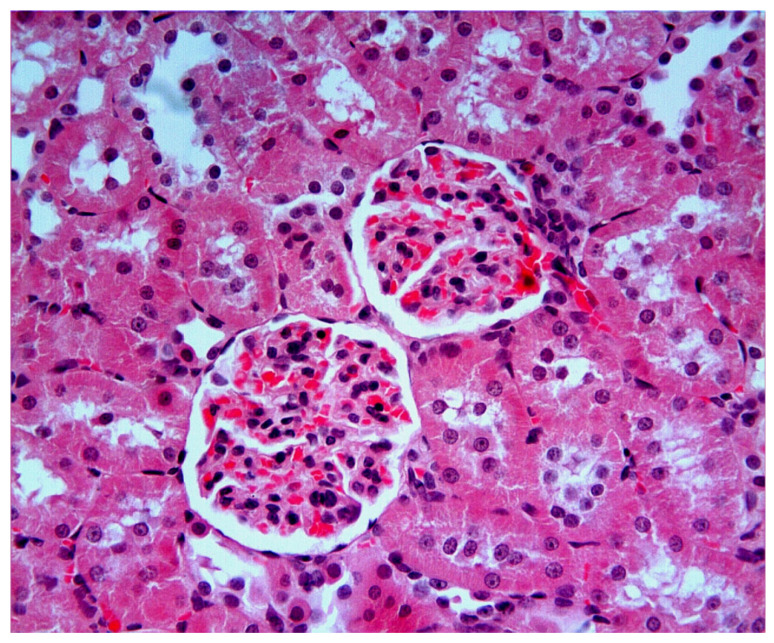
Control group. Kidney tissue from the Control group showed a normal histological appearance. Glomerular and tubular structures were found to be normal. H-E; ×40.

**Figure 5:**
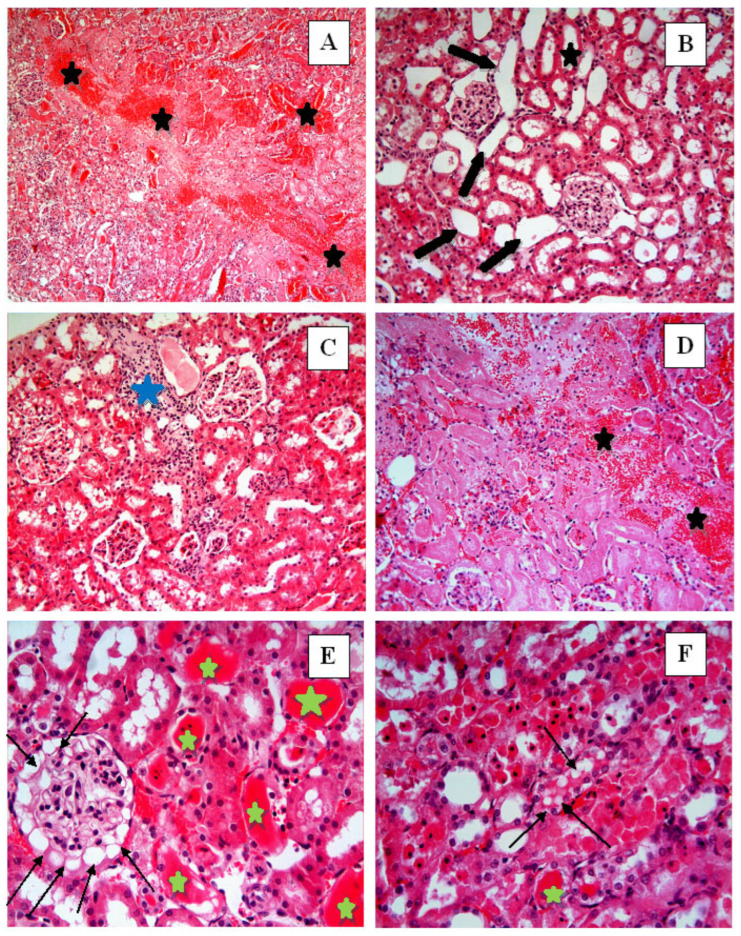
I/R group. In the I/R group, hemorrhage (black star) (**A**,**D**), tubular lumen dilation (thick black arrows) (**B**), inflammatory cell infiltration (blue star) (**C**), tubular and glomerular degeneration (**B**–**E**), vacuolization in glomeruli (thin black arrows) (**E**), vacuolization in tubular epithelial cells (thin black arrows) (**F**), epithelial cell atrophy and shedding of tubular epithelial cells (**E**,**F**), and accumulation of material in the tubular lumen (green star) (**E**,**F**) were observed. (**A**): H-E, ×10; (**B**–**D**): H-E, ×20; (**E**,**F**): H-E, ×40.

**Figure 6:**
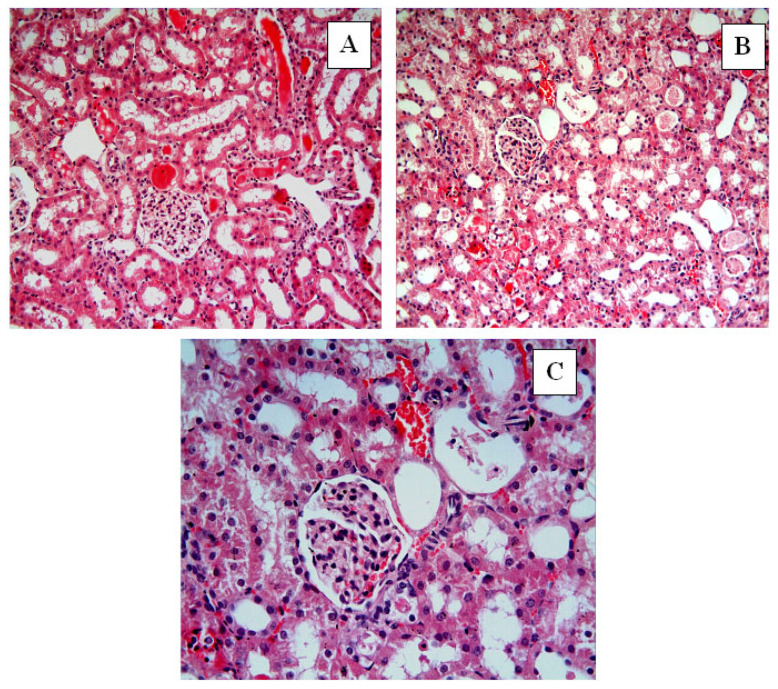
I/R + Nat 50. In the I/R + Nat 50 group, a small amount of hemorrhage (**A**–**C**), tubular dilation (**A**–**C**), and a small amount of material accumulation in the tubule lumen were observed. (**A**,**B**): H-E, ×20; (**C**): H-E, ×40.

**Figure 7:**
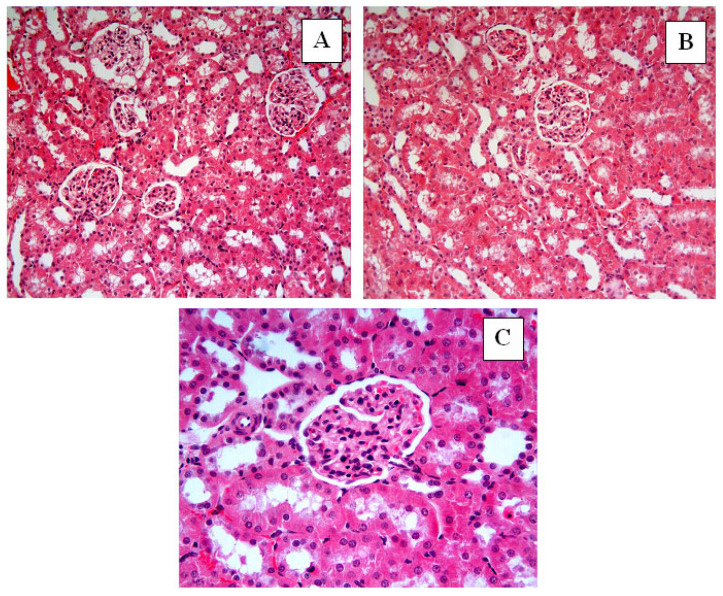
I/R + Nat 100. The I/R + Nat 100 group showed significantly less damage compared to the I/R group. More regular tubular and glomerular structures were observed, along with minimal hemorrhage and tubular dilation (**A**–**C**). (**A**,**B**): H-E, ×20; (**C**): H-E, ×40.

In the Control group (Figure 4), renal tissue exhibited intact histological architecture with well-organized glomeruli and tubules. In the I/R group, hemorrhage (black star) (Figure 5A,D), tubular lumen dilatation (thick black arrows) (Figure 5B), inflammatory cell infiltration (blue star) (Figure 5C), tubular and glomerular degeneration (Figure 5B–E), vacuolization in glomeruli (thin black arrows) (Figure 5E), vacuolization in tubular epithelial cells (thin black arrows) (Figure 5F), epithelial cell atrophy and shedding of tubular epithelial cells (Figure 5E,F), and accumulation of material in the tubular lumen (green star) (Figure 5E,F) were observed. In the I/R + Nat 50 group, a small amount of hemorrhage (Figure 6A–C), tubular dilation (Figure 6A–C), and a small amount of material accumulation in the tubule lumen were observed. The I/R + Nat 100 group showed significantly less damage compared to the I/R group. More regular tubular and glomerular structures were observed, along with minimal hemorrhage and tubular dilation (Figure 7A–C).

Compared with the I/R group, histopathological injury scores were reduced by 15.8% in the I/R + Nat 50 group and 34.4% in the I/R + Nat 100 group, demonstrating substantial structural preservation of renal tissue following Nat treatment.

Histopathological scores are given in Table 1.

**Figure 8:**
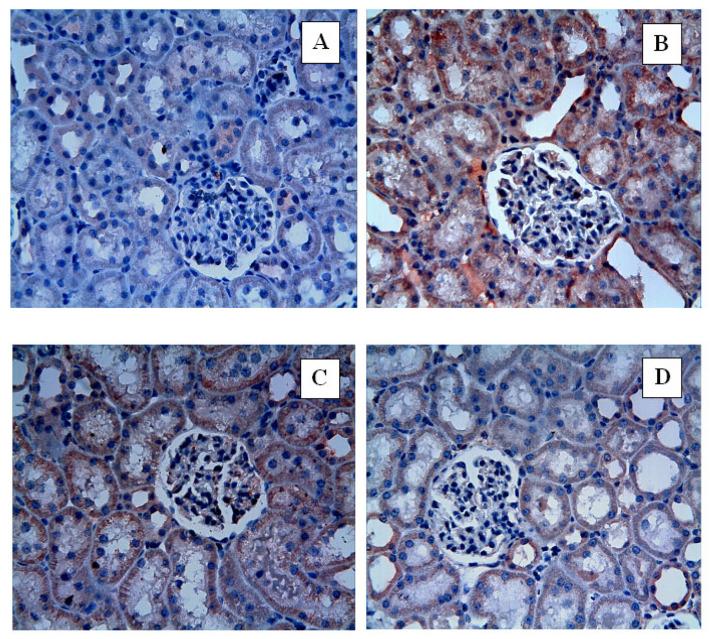
Immunohistochemical staining of caspase-3 in renal sections (Control (**A**), I/R group (**B**), I/R + Nat 50 (**C**), and I/R + Nat 100 (**D**)). (**A**–**D**): ×40.

In caspase-3 immunohistochemical analysis, no caspase-3 immunoreactivity was detected in the Control group, whereas the I/R group showed intense caspase-3 immunoreactivity. Nat treatment markedly reduced caspase-3 immunoreactivity, with the most pronounced reduction observed in the 100 mg/kg group (*p* < 0.0001; Figure 8, Table 2). Compared with the I/R group, caspase-3 immunoreactivity scores were reduced by 32.2% in the I/R + Nat 50 group and 39.3% in the I/R + Nat 100 group, indicating a marked attenuation of apoptosis following Nat treatment.

Caspase-3 staining scores are given in Table 2.

## 3. Discussion

During I/R injury, hypoxia followed by reperfusion leads to excessive ROS production, which suppresses endogenous antioxidant defense systems [19]. This exacerbates oxidative damage at the cellular level and impairs tissue function. Experimental studies have demonstrated that following I/R injury, antioxidant parameters such as SOD, CAT, and GSH decrease, while lipid peroxidation products such as TBARS significantly increase [20]. These biochemical alterations are regarded as important indicators of early cellular injury as well as functional impairment. In line with these experimental findings, in our study, these parameters were also evaluated to investigate the potential protective role of Nat against oxidative stress [21].

In our study, serum BUN levels were significantly increased in the renal I/R group compared to the Control group, consistent with renal dysfunction following I/R injury. This increase may be related to changes in urea processing by the kidney and metabolic stress during injury; however, the precise mechanism underlying this elevation remains unclear. The literature also reports that BUN levels are significantly elevated in I/R models and serve as a key indicator of renal dysfunction [22]. In the Nat-pretreated groups (50 mg/kg and 100 mg/kg), this I/R-induced elevation was significantly attenuated. However, the absence of a distinct difference between the two doses suggests that the effect of Nat may plateau beyond a certain threshold. Similarly, serum creatinine levels were significantly increased in the I/R group compared with the Control group. This elevation is considered a biochemical indicator of impaired glomerular filtration and tubular injury. In the Nat-pretreated groups, this increase was markedly reduced, supporting the possibility that Nat may alleviate I/R-induced renal dysfunction. In the renal I/R group, serum TNF-α levels were significantly increased compared to the Control group. This increase indicates strong activation of the inflammatory response during the I/R process. Similarly, Bayoumi et al. reported elevated TNF-α levels in rats following renal I/R injury, which were associated with renal dysfunction [23]. The significant reduction in TNF-α levels in the Nat-treated groups is consistent with the anti-inflammatory effects of the drug. This attenuation may be attributed to the modulation of inflammatory mediators by Nat, as suggested in previous experimental models. In addition to TNF-α, IL-1β is another key pro-inflammatory cytokine that plays a central role in the inflammatory cascade during renal I/R injury. Serum IL-1β levels were also significantly increased in the I/R group compared to the Control group. This finding demonstrates that the inflammatory process is activated in renal tissue. In line with our results, Fathy et al. reported elevated IL-1β levels in rats following renal I/R injury, which were associated with functional impairment [24]. The reduction in IL-1β levels in the Nat-treated groups may be partly related to the anti-inflammatory actions of Nat, which have been demonstrated in earlier studies. The activation of pro-inflammatory cytokines such as TNF-α and IL-1β is closely linked to mitochondrial dysfunction and excessive ROS production during I/R injury. During I/R injury, mitochondrial dysfunction leads to the production of superoxide radicals, which enhance oxidative stress at the cellular level and exacerbate tissue damage. SOD, one of the major antioxidant defense mechanisms of the organism, plays a critical role in detoxifying these superoxide radicals [7,25]. Accordingly, alterations in SOD activity are considered a sensitive indicator of oxidative stress severity in renal I/R models [26]. In this study, SOD levels were significantly reduced in the I/R group. Nat, an antidiabetic agent, significantly reversed the decrease in SOD levels caused by I/R. The dose-dependent increase in SOD activity observed in the Nat-treated groups indicates that the drug reduces oxidative damage and highlights its potential antioxidant effect. Other antioxidant enzymes such as CAT play a complementary role in maintaining redox balance during renal I/R injury. CAT is a fundamental antioxidant enzyme that reduces intracellular oxidative load by converting ROS such as hydrogen peroxide into water and oxygen. In the renal I/R model, the rapid accumulation of ROS immediately after reperfusion suppresses endogenous antioxidant defenses, resulting in diminished CAT activity [27]. In our study, this suppression was evident in the I/R group. Conversely, CAT activity increased markedly in the Nat-treated groups, indicating a potential role of the drug in mitigating oxidative stress. Notably, the highest CAT activity detected in the 100 mg/kg Nat group supports the close relationship between enhanced antioxidant capacity and improved tissue protection. In addition to enzymatic antioxidants such as SOD and CAT, non-enzymatic antioxidant systems also play a pivotal role in cellular defense against oxidative stress. GSH is one of the major non-enzymatic antioxidants responsible for maintaining intracellular redox balance and is among the most affected targets of I/R-induced oxidative stress. The literature reports significant reductions in renal GSH levels following I/R in rats, associated with both increased ROS production and rapid GSH consumption [28,29,30]. In the Nat-treated groups, GSH levels were significantly increased compared to the I/R group. Both 50 mg/kg and 100 mg/kg doses of Nat produced similar increases, suggesting that the drug may not provide additional benefit beyond a certain threshold dose. The increase in renal GSH levels observed following nateglinide treatment may reflect partial restoration of the intracellular glutathione pool through multiple complementary mechanisms. Improvement of antioxidant enzyme activities and reduction in lipid peroxidation may decrease ROS-mediated GSH consumption, thereby preserving reduced glutathione availability. In addition, enhanced regeneration of GSH from its oxidized form (GSSG), potentially mediated by glutathione reductase-dependent recycling and improved cellular redox balance, may contribute to this effect. A compensatory increase in de novo glutathione synthesis via redox-sensitive pathways cannot be excluded; however, the absence of direct measurements of glutathione-related enzymes and peroxide metabolism limits definitive mechanistic interpretation. Therefore, the observed increase in GSH should be interpreted as indicative of improved redox homeostasis rather than confirmation of a specific molecular mechanism.

In our study, TBARS levels were significantly elevated in the I/R group compared with controls, consistent with previous findings demonstrating marked lipid peroxidation following renal ischemia [20]. Nat treatment markedly reduced TBARS levels at both doses, indicating attenuation of ROS-induced lipid damage. The greater reduction observed in the 100 mg/kg group suggests a dose-dependent improvement in membrane integrity, supporting the biochemical evidence of enhanced antioxidant defense. However, although Nat treatment significantly improved antioxidant enzyme activities and reduced lipid peroxidation, TBARS levels did not fully return to control values, indicating that oxidative stress was attenuated but not completely normalized following renal I/R injury. Therefore, the observed renoprotective effects should be interpreted as partial restoration of redox balance rather than complete suppression of oxidative stress.

Although classical biochemical markers such as SOD, CAT, GSH, and TBARS remain widely used indicators of oxidative stress, current concepts in redox biology emphasize the dynamic balance between oxidant production, antioxidant defense systems, and cellular adaptive responses. Therefore, the observed biochemical alterations in the present study should be interpreted as reflecting modulation of overall redox homeostasis rather than complete normalization of oxidative status.

These biochemical findings were further corroborated by histopathological observations, demonstrating that Nat-mediated antioxidant effects translated into structural renal protection. In light of these data, histopathological findings further support the dose-dependent renoprotective effect of Nat in renal I/R injury. While limited improvement was observed at the lower dose, the higher dose preserved structural integrity and markedly reduced apoptosis. Severe histopathological changes were observed in the I/R group. Notably, tubular epithelial dilatation, degeneration, hemorrhage, material accumulation, and inflammatory cell infiltration were evident. Structural disruption of glomeruli, vacuolization, and cellular atrophy were identified as being associated with excessive ROS production during reperfusion.

Histopathological evaluation further supported the biochemical findings, demonstrating that renal I/R injury caused marked structural alterations including tubular degeneration, epithelial desquamation, and inflammatory cell infiltration. Nat treatment attenuated these morphological abnormalities and preserved renal tissue architecture, particularly at the higher dose. Consistent with histological improvement, immunohistochemical analysis revealed a significant reduction in caspase-3 immunoreactivity in Nat-treated groups, indicating suppression of apoptosis. It should be noted that the antibody used for caspase-3 immunohistochemical staining in this study (Neomarkers, Rabbit PAb, RB-1197-P) detects total caspase-3, including both the inactive precursor (procaspase-3) and the cleaved active forms. Therefore, the observed immunoreactivity reflects overall caspase-3 expression rather than the specific detection of activated caspase-3. Despite this limitation, the reduced caspase-3 immunoreactivity observed in the Nat-treated groups may indicate attenuation of apoptotic processes following renal ischemia–reperfusion injury. These findings suggest that the renoprotective effects of Nat are not limited to biochemical modulation but are also reflected at the tissue level through structural preservation and reduced apoptotic activity.

## 4. Materials and Methods

### 4.1. Experimental Animals

This study was conducted with the approval of the Local Animal Ethics Committee of Inonu University Faculty of Medicine (protocol number 2024/15-2). The animal experiments were carried out at the Inonu University Experimental Animal Production and Research Center, while the analyses were performed in the laboratories of the Departments of Physiology and Histology & Embryology. A total of 40 male Sprague Dawley rats weighing 250–300 g were used. Animals were housed individually under controlled conditions (20–22 °C, 50–60% humidity, 12 h light/dark cycle) with free access to standard pellet chow and tap water.

### 4.2. Ethical Approval and Animal Welfare

All experimental procedures involving animals were reviewed and approved by the Local Animal Ethics Committee of Inonu University, Faculty of Medicine (Malatya, Türkiye) (Approval No.: 2024/15-2; Date: 26 August 2024).

This study was conducted in accordance with the ARRIVE guidelines and complied with the European Union Directive 2010/63/EU for animal experiments and the NIH Guide for the Care and Use of Laboratory Animals.

### 4.3. Experimental Procedure and Modeling

The rats were randomly assigned to four groups (*n* = 10): Control, I/R, I/R + Nat 50 mg/kg, and I/R + Nat 100 mg/kg. Rats were anesthetized with 70 mg/kg ketamine and 8 mg/kg xylazine administered intraperitoneally [31]. No surgical or pharmacological intervention was applied to the Control group. In the I/R group, 1 mL of physiological saline (vehicle) was administered orally, followed by a 1 h waiting period [32]. Subsequently, both renal pedicles were clamped for 45 min to induce ischemia, and the clamps were then removed to allow 24 h of reperfusion [33,34].

In the Nat-pretreated groups, 50 mg/kg and 100 mg/kg Nat were administered via oral gavage, and 1 h after administration, the same I/R protocol (45 min of ischemia + 24 h of reperfusion) was performed. At the end of the experimental period, all animals were decapitated, and blood and renal tissues were collected. TBARS and GSH levels, as well as SOD and CAT enzyme activities, were measured in the right kidney, while serum BUN, creatinine, TNF-α, and IL-1β levels were analyzed. The left kidney tissues were used for histological and immunohistochemical evaluations.

#### Nateglinide Source and Preparation

Nat used in this study was prepared by suspending the calculated doses (50 mg/kg and 100 mg/kg) in 15 mL of sterile physiological saline (0.9% NaCl) to obtain a homogeneous gavage solution. The suspension was thoroughly vortexed until complete homogenization was achieved, and fresh preparations were administered via oral gavage one hour before ischemia [35].

Serum samples were obtained by centrifugation and stored at −80 °C for biochemical analyses, while renal tissues for histological evaluation were fixed in 10% neutral-buffered formalin [36].

**Figure 9:**
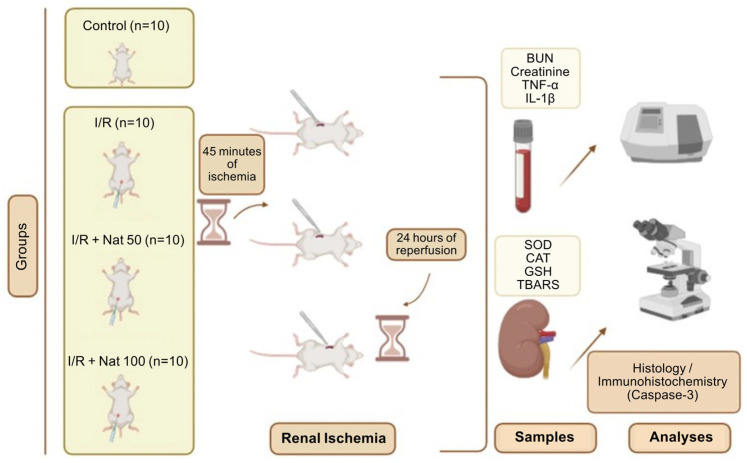
Schematic representation of the experimental design (I/R: ischemia/reperfusion; Nat: nateglinide; BUN: blood urea nitrogen; creatinine: creatinine; TNF-α: tumor necrosis factor-alpha; IL-1β: interleukin-1 beta; SOD: superoxide dismutase; CAT: catalase; GSH: glutathione; TBARSs: thiobarbituric acid reactive substances).

The experimental flowchart is shown in Figure 9.

### 4.4. Biochemical Analyses

For biochemical analyses, kidney tissues were rinsed with ice-cold physiological saline to remove blood residues and homogenized in ice-cold phosphate buffer (50 mM, pH 7.4) using a glass–Teflon homogenizer to obtain a 10% (*w*/*v*) tissue homogenate. Homogenization procedures were carried out on ice to prevent enzymatic degradation. The homogenates were centrifuged at 10.000× *g* for 15 min at 4 °C, and the resulting supernatants were collected and used for the determination of oxidative stress parameters.

#### 4.4.1. Blood Urea Nitrogen and Creatinine

Serum BUN and creatinine levels were measured using an automated biochemistry analyzer (Olympus Instruments, Tokyo, Japan).

#### 4.4.2. Inflammatory Markers

Serum TNF-α and IL-1β levels were determined using commercially available ELISA kits (Rat TNF-α ELISA kit, Elabscience Biotechnology Inc., Cat. No: E-EL-R2856; Rat IL-1β ELISA kit, Elabscience Biotechnology Inc., Cat. No: E-EL-R0012; Houston, TX, USA) according to the manufacturer’s protocol. The wells were pre-coated with specific antibodies. After sample loading, antigen–antibody binding occurred, followed by incubation with a biotinylated detection antibody and HRP conjugate. Following the washing steps, a substrate solution was added, producing a blue color that turned yellow with the addition of the stop solution.

#### 4.4.3. Superoxide Dismutase

SOD activity was evaluated according to the method of Sun et al. [37], which is based on the inhibition of Nitro Blue Tetrazolium (NBT) reduction by superoxide radicals generated from the xanthine/xanthine oxidase system. One unit of SOD activity was defined as the amount of enzyme required to inhibit the NBT reduction rate by 50%. Results were expressed as U/mg protein. Total superoxide dismutase (SOD) activity was measured according to the method of Sun et al. This assay determines overall SOD activity and does not differentiate between individual SOD isoforms (Cu/Zn-SOD, Mn-SOD, or extracellular SOD).

#### 4.4.4. Catalase

CAT activity was measured according to the method of Aebi [38]. The decrease in absorbance of an H_2_O_2_ solution prepared in phosphate buffer was monitored spectrophotometrically at 240 nm. Absorbance was recorded at 15 s intervals for 5 min.

#### 4.4.5. Glutathione

Renal GSH levels were determined according to the method of Ellman [39]. Ten percent trichloroacetic acid was added to the homogenates, and after centrifugation, the supernatant was mixed with 5,5′-dithiobis-2-nitrobenzoic acid. Absorbance was measured at 410 nm and calculated using a standard curve.

#### 4.4.6. Thiobarbituric Acid Reactive Substance Levels

Renal TBARS levels were measured according to the method of Mihara and Uchiyama [40]. Homogenate samples were treated with 1% phosphoric acid and 0.6% thiobarbituric acid and incubated at 90 °C for 45 min. After cooling, extraction was performed with n-butanol. Following centrifugation, the absorbance of the organic upper phase was measured at 535 nm. TBARS concentrations were calculated using a 1,1,3,3-tetraethoxypropane standard curve and expressed as nmol/g tissue.

#### 4.4.7. Protein Measurement

Protein concentration was determined using the Lowry method [41]. Bovine serum albumin was used as the standard, and results were calculated from a standard curve and expressed as mg/mL.

### 4.5. Histological and Immunohistochemical Analyses

For light microscopic evaluation, kidney samples were fixed in 10% formalin. The tissue samples were processed by routine tissue techniques and were embedded in paraffin. Paraffin-embedded specimens were cut into 5 μm thick sections, mounted on slides and stained with hematoxylin–eosin (H-E). Sections were examined under a Leica DFC280 light microscope by Leica Q Win and Image Analysis System (Leica Micros Imaging Solutions Ltd.; Cambridge, UK).

For immunohistochemical analysis, thick sections were mounted on polylysine-coated slides. After rehydrating, samples were transferred to citrate buffer (pH 7.6) and heated in a microwave oven for 20 min. After cooling for 20 min at room temperature, the sections were washed with phosphate-buffered saline (PBS). Then sections were kept in 0.3% H_2_O_2_ for 7 min and washed afterward with PBS. Sections were incubated with primary rabbit-polyclonal Caspase-3 antibody (Abcam, Ab4051; Cambridge, UK) for two hours. They were then rinsed in PBS and incubated with biotinylated goat antipolyvalent for 10 min and streptavidin peroxidase for 10 min at room temperature. Staining was completed with chromogen + substrate for 15 min, and slides were counterstained with Mayer’s hematoxylin for 1 min, rinsed in tap water, and dehydrated. The Caspase-3 kit (Neomarkers, Rabbit PAb, RB-1197-P; Fremont, CA, USA) was used according to the manufacturer’s instructions [42].

Histopathological damage scores were calculated based on these findings. The severity of renal injury was evaluated using a semi-quantitative scoring system as follows: 0 (none), 1 (mild), 2 (moderate), and 3 (severe). Caspase-3 immunohistochemical staining was evaluated semi-quantitatively according to staining intensity as follows: 0–25% = 0, 25–50% = 1, 50–75% = 2, and 75–100% = 3.

### 4.6. Statistical Analyses

Data were analyzed using SPSS 22.0 software (SPSS Inc., Chicago, IL, USA). Results for biochemical parameters were expressed as mean ± standard deviation (SD). Semi-quantitative histopathological and immunohistochemical scores were expressed as median (minimum–maximum). The Shapiro–Wilk test was used to assess normality. Group comparisons were performed using the Kruskal–Wallis H test, and when significant differences were detected, pairwise comparisons were performed using the Bonferroni-corrected Mann–Whitney U test. A *p*-value < 0.05 was considered statistically significant.

## 5. Conclusions

In conclusion, the biochemical, histopathological, and immunohistochemical findings of the present study indicate that Nat exerts a notable renoprotective effect against renal I/R injury in rats. Our results suggest, for the first time, that Nat may protect renal tissue through antioxidant, anti-inflammatory, and anti-apoptotic mechanisms. Furthermore, the findings demonstrated a dose-dependent improvement, with the 100 mg/kg dose providing the most pronounced tissue protection. While several antidiabetic agents have been investigated for their renoprotective effects in AKI, experimental evidence regarding Nat in renal I/R injury remains limited. The present study demonstrates that Nat exerts early renoprotective effects within an acute reperfusion period, suggesting a potential role as a short-term prophylactic or adjunctive pharmacological strategy rather than a conventional antidiabetic intervention. Although several pharmacological agents have been evaluated for the prevention of I/R-induced injury, further comprehensive studies are required to determine whether the protective effects of Nat extend to other organ systems and to better understand its translational potential.

## 6. Limitations

The present study has several limitations. First, only male rats were used, which limits the ability to generalize the findings to both sexes, as renal I/R injury and drug responses may exhibit sex-related differences. Second, although the study demonstrated consistent biochemical, histopathological, and immunohistochemical improvements with Nat treatment, the specific molecular pathways underlying these protective effects were not investigated. Evaluation of additional hydrogen peroxide-detoxifying systems, such as glutathione peroxidase activity and total hydroperoxide levels, may further clarify the underlying mechanisms and should be addressed in future studies. Additionally, only two doses of Nat were tested, and dose–response relationships beyond these levels remain unknown. Future studies employing both sexes, broader dosing strategies, and detailed mechanistic analyses may provide a more comprehensive understanding of the renoprotective potential of Nat in renal I/R injury.

## Figures and Tables

**Table 1:** Histopathological analysis results.

Groups	Histopathological Score (Med (Min–Max))
Control	(1 (0–2)) ^a^
I/R	(2 (1–3)) ^b^
I/R + Nat 50	(2 (1–3)) ^c^
I/R + Nat 100	(1 (0–3)) ^d^

Data are presented as median (min–max). Lowercase letters (a–d) in the same column indicate statistically significant differences between groups (*p* < 0.0001).

**Table 2:** Caspase-3-positive staining score table.

Groups	Staining Score (Med (Min–Max))
Control	(1 (0–2)) ^a^
I/R	(3 (1–3)) ^b^
I/R + Nat 50	(2 (1–3)) ^c^
I/R + Nat 100	(2 (1–3)) ^c^

Data are expressed as median (min–max). Different lowercase letters (a–c) in the same column indicate statistically significant differences between groups (*p* < 0.0001).

## Data Availability

The datasets generated and/or analyzed during the current study are available from the corresponding author upon reasonable request.

## Abbreviations

The following abbreviations are used in this manuscript:

AKI	Acute kidney injury
BUN	Blood urea nitrogen
CAT	Catalase
GSH	Glutathione
H&E	Hematoxylin and eosin
I/R	Ischemia/reperfusion
IL-1β	Interleukin-1 beta
TBARS	Thiobarbituric acid reactive substances
Nat	Nateglinide
NBT	Nitro blue tetrazolium
PBS	Phosphate-buffered saline
ROS	Reactive oxygen species
SOD	Superoxide dismutase
TNF-α	Tumor necrosis factor-alpha
